# An epidemiological analysis of Acute Flaccid Paralysis (AFP) surveillance in Kenya, 2016 to 2018

**DOI:** 10.1186/s12879-020-05319-6

**Published:** 2020-08-18

**Authors:** Brook Tesfaye, Alieu Sowe, Ngina Kisangau, John Ogange, Stephen Ntoburi, Irene Nekar, Charles Muitherero, Yaya Camara, Carolyne Gathenji, Daniel Langat, Kibet Sergon, Hilary Limo, Rosemary Nzunza, Shem Kiptoon, David Kareko, Iheoma Onuekwusi

**Affiliations:** 1 World Health Organization, Kenya Country Office, United Nations Office in Nairobi (UNON), Gigiri Complex, Block U, Nairobi, Kenya; 2 Polio Surge Consultant, African Field Epidemiology Network, Nairobi, Kenya; 3 Horn of Africa Polio Eradication Coordination Office, Nairobi, Kenya; 4 Division of Disease Surveillance and Response, Ministry of Health, Nairobi, Kenya; 5 Kenya Medical Research Institute, Center for Virus Research, Ministry of Health, Nairobi, Kenya

**Keywords:** Polio, Acute Flaccid Paralysis (AFP), Surveillance, Immunization, Kenya

## Abstract

**Background:**

The poliovirus has been targeted for eradication since 1988. Kenya reported its last case of indigenous Wild Poliovirus (WPV) in 1984 but suffered from an outbreak of circulating Vaccine-derived Poliovirus type 2 (cVDPV2) in 2018. We aimed to describe Kenya’s polio surveillance performance 2016–2018 using WHO recommended polio surveillance standards.

**Methods:**

Retrospective secondary data analysis was conducted using Kenyan AFP surveillance case-based database from 2016 to 2018. Analyses were carried out using Epi-Info statistical software (version 7) and mapping was done using Quantum Geographic Information System (GIS) (version 3.4.1).

**Results:**

Kenya reported 1706 cases of AFP from 2016 to 2018. None of the cases were confirmed as poliomyelitis. However, 23 (1.35%) were classified as polio compatible. Children under 5 years accounted for 1085 (63.6%) cases, 937 (55.0%) cases were boys, and 1503 (88.1%) cases had received three or more doses of Oral Polio Vaccine (OPV). AFP detection rate substantially increased over the years; however, the prolonged health workers strike in 2017 negatively affected key surveillance activities. The mean Non-Polio (NP-AFP) rate during the study period was 2.87/ 100,000 children under 15 years, and two adequate specimens were collected for 1512 (88.6%) AFP cases. Cumulatively, 31 (66.0%) counties surpassed target for both WHO recommended AFP quality indicators.

**Conclusions:**

The performance of Kenya’s AFP surveillance system surpassed the minimum WHO recommended targets for both non-polio AFP rate and stool adequacy during the period studied. In order to strengthen the country’s polio free status, health worker’s awareness on AFP surveillance and active case search should be strengthened in least performing counties to improve case detection. Similar analyses should be done at the sub-county level to uncover underperformance that might have been hidden by county level analysis.

## Background

Poliomyelitis, commonly known as Polio, is an infectious disease caused by the Poliovirus [1, 2]. There are three serotypes of the Wild Poliovirus (WPV); type 1, 2 and 3 – types 2 and 3 have been eradicated. The Poliovirus is transmitted from person to person primarily through contaminated fecal matter entering the oral route [1, 3]. It can also be transmitted in rare cases through saliva [3, 4]. Human beings are the only known reservoir for the poliovirus [5]. The Poliovirus replicates in the intestine of its human host and consequently spreads to the central nervous system [3, 6].

The burden of polio disease is higher among children under 5 years of age [3, 7, 8]. Poliovirus infection causes irreversible paralytic disease, presenting as Acute Flaccid Paralysis (AFP), in 1 per 200 to 1 per 1000 cases [9] with a Case Fatality Rate (CFR) of 5 to 10% [6]. Poliomyelitis has no cure [10]. However, it is possible to prevent the disease through vaccination [10, 11].

The forty-first World Health Assembly (WHA) in Geneva Switzerland in May 1988 was a breakthrough for the commencement of The Global Polio Eradication Initiative (GPEI) led by national governments in partnership with the World Health Organization (WHO), Rotary Foundation, Bill and Melinda Gates Foundation (BMGF), United States Centers for Disease Control (US CDC), and United Nations Children’s Fund (UNICEF) [5, 8, 12]. The initiative targeted to eradicate the poliovirus worldwide by the year 2000 using four proven strategies [13]. These are: 1) maintaining high population immunity using OPV and IPV through the Expanded Programme on Immunization (EPI), 2) detect and interrupt circulation of all suspected cases of Poliomyelitis through sensitive AFP surveillance, 3) Supplemental Immunization Activities (SIAs), and 4) mop-up campaigns [14, 15]. A sensitive AFP surveillance system is central to the overall polio eradication initiative [4, 16]. The GPEI has a set of performance indicators to monitor progress and evaluate performance of countries [1]. These global efforts have reduced the number of WPV cases by more than 99%: from an estimated 350,000 of WPV cases from 125 countries in 1988 to only 33 cases by the end of 2018 [17, 18]. Since August 2016, WPV cases have not been detected outside Afghanistan and Pakistan [17–19].

In Kenya, under the Ministry of Health (MOH), the department of Disease Surveillance and Response (DDSR) in collaboration with the National Vaccine and Immunization Program (NVIP) collaborate on the Polio eradication activities. The activities aim to ensure the certification of the country as free of indigenous wild poliovirus in the shortest possible time and maintain polio free status. The last case of WPV (non-indigenous) in Kenya was detected in 2013, with a paralysis onset date of 14 July 2013 [20]. This was an imported AFP case from neighboring Somalia [20].

Describing characteristics of AFP cases and assessing performance towards set goals are of great importance in providing quantitative evidence to support policymakers in making informed decisions. For this reason, this study aims to describe Kenya’s AFP surveillance performance from 2016 to 2018 based on the WHO recommended epidemiological performance indicators [1] for AFP surveillance.

## Methods

### Study setting

**Fig. 1:**
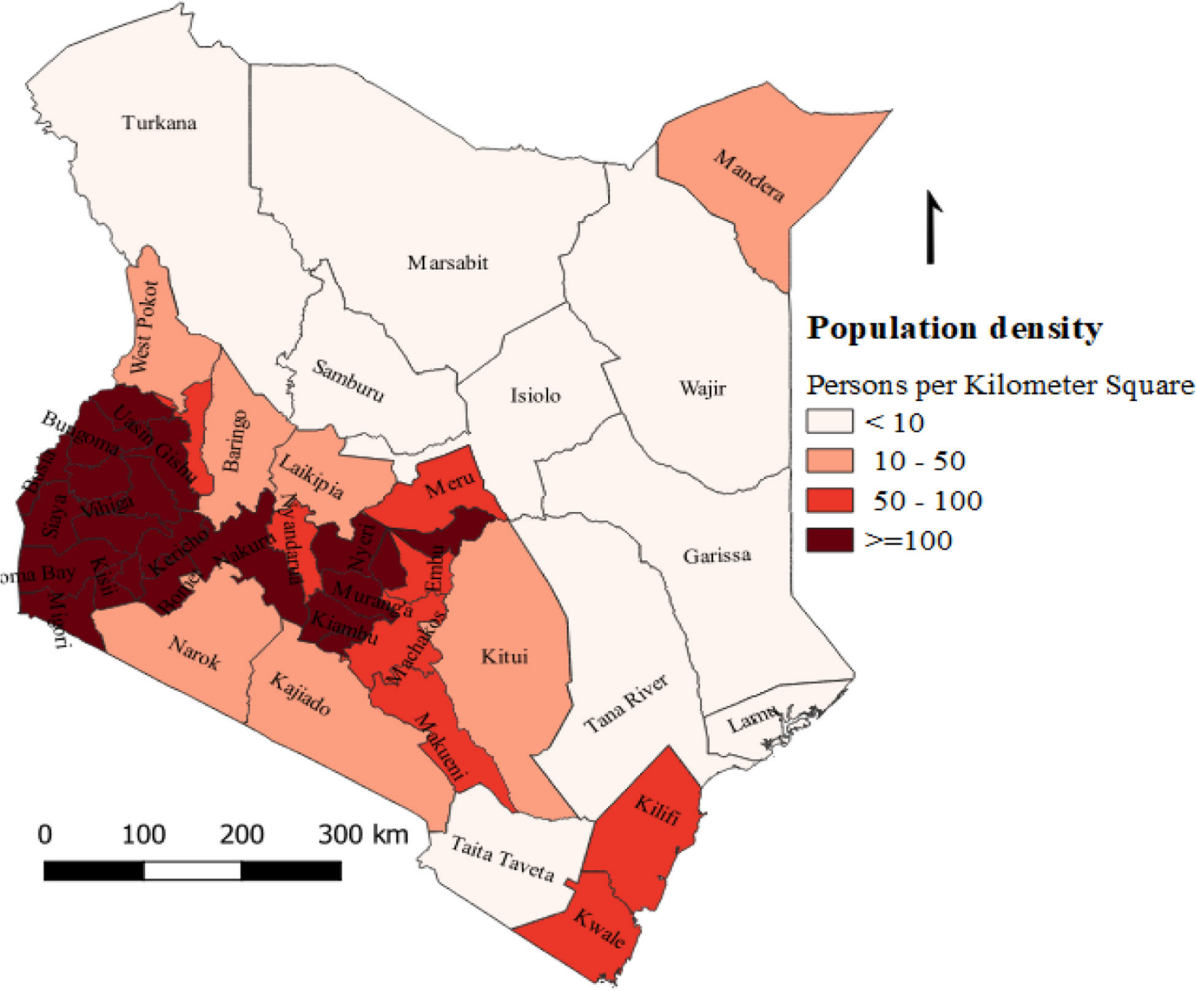
Under-fifteen population density by county, Kenya, 2019

Kenya is located in East Africa and the country has forty-seven semi-autonomous counties, which are further divided into sub counties. In 2018, Kenya had an estimated total population of over 52 million of which nearly half (46%) were children under the age of 15 years [21]. The under-fifteen population density by county was depicted in Fig. 1.

### Study design and period

Retrospective secondary data analysis was performed on the AFP surveillance case-based database from January 2016 to December 2018.

### AFP surveillance

The WHO defined an AFP case as a child < 15 years of age presenting with sudden onset of floppy paralysis or muscle weakness due to any cause, or any person of any age with paralytic illness if poliomyelitis is suspected by a clinician [1]. Acute Flaccid Paralysis occurring to a person highly suspected for polio based on the clinical suspicion of poliomyelitis or who has either received less than three doses of polio vaccine, or has recently travelled to an endemic country (having returned up to 35 days before the onset of paralysis) or belonging to a high risk group [22, 23] is considered a hot AFP case. A country’s surveillance system is expected to detect at least one case of AFP in every 100,000 children under 15 years of age - depending on the polio eradication context (certified Polio free, endemic or outbreak) of the country [16]. All health care workers and other members of the surveillance network are expected to notify Sub-County Disease Surveillance Coordinators (SCDSCs) of all cases that meet the AFP case definition within 24 h. However, hot AFP cases should be reported immediately using the fastest possible means., All reported AFP cases should be investigated within 48 h. If a case is believed to meet the AFP case definition, the required sections of a dedicated case investigation form (MOH 502) are filled and two stool samples are collected 24 to 48 h apart and kept in the reverse cold chain. Then the SCDSCs transport the filled case investigation form and the specimen to the WHO accredited Polio laboratory at Kenya Medical Research Institute (KEMRI) for Poliovirus isolation and intra-typic differentiation. The specimen should be transported to the KEMRI laboratory within 72 h. If two stool specimens are not collected within 14 days of onset of paralysis or the stools do not reach the laboratory in good condition, one-stool sample is collected from each of three close contacts of the case, preferably from children under the age of 5 years [18]. If a sub county with more than 100,000 under 15 years population is “Silent” that is, not detected a single case of AFP for a period of 6 months to 1 year, stool specimens are supposed to be collected from three healthy children from the community [1, 18]. A National Polio Expert Committee (NPEC), comprising of pediatricians, neurologists, epidemiologists and government officials, is responsible for the final classification of AFP cases upon reception of appropriate documentation on a quarterly basis.

### AFP surveillance indicators

The GPEI developed several performance indicators for monitoring the performance (sensitivity and quality) of AFP surveillance programs [18, 24]. However, two key indicators are most commonly used to monitor AFP surveillance sensitivity and quality [25]. These are the Non-Polio Acute Flaccid Paralysis (NP-AFP) rate and the proportion of AFP cases with adequate stool specimens [18]. The target for Non-Polio AFP cases per 100,000 population under the age of 15 years is at least 1 (depending on the country’s polio context) and the recommended minimum proportion of reported AFP cases that should have adequate stool specimens is 80% [1]. An AFP stool sample is considered adequate if two stool specimens are collected 24 h to 48 h within 14 days of paralysis onset, of sufficient quantity (8–10 g) and specimens reached the reference laboratory in good condition [1]. Good stool condition is defined as specimen that reached that laboratory within 72 h of collection without desiccation or leakage, with evidence of reverse cold chain maintenance during transportation to the laboratory, and with appropriate documentation [1, 26]. For each AFP case with inadequate specimen, one stool specimen should be collected from each of three contacts. Contacts of AFP cases are children under 15 years of age who had direct contact with AFP case within 1 week before or 2 weeks after onset of paralysis [18], An AFP case is classified as polio compatible if the stool specimens were not adequate to rule out the poliovirus and the case had developed residual paralysis either after 60 days follow up, died within 60 days or was lost to follow up ahead of investigation to rule out poliomyelitis as causative agent [18, 27].

### Data collection procedures and analysis

The database for this study is the AFP surveillance case-based database obtained from the WHO Kenya country office upon justified request. The database was retrieved in Microsoft (MS) Office Access file format. Statistical data analysis was conducted using Epi-Info statistical software (version 7; CDC, Atlanta, United States) and Quantum GIS (version 3.4.1) was used for spatial analysis. Descriptive analyses were performed to describe the epidemiology of reported AFP cases in Kenya and statistics based on the WHO recommended performance indicators for AFP surveillance were generated [28]. Mapping was done to visualize surveillance performance and distribution of AFP cases by location. Results of the study are presented in the form of tables and maps.

## Results

### Demographic characteristics and clinical history

**Fig. 2:**
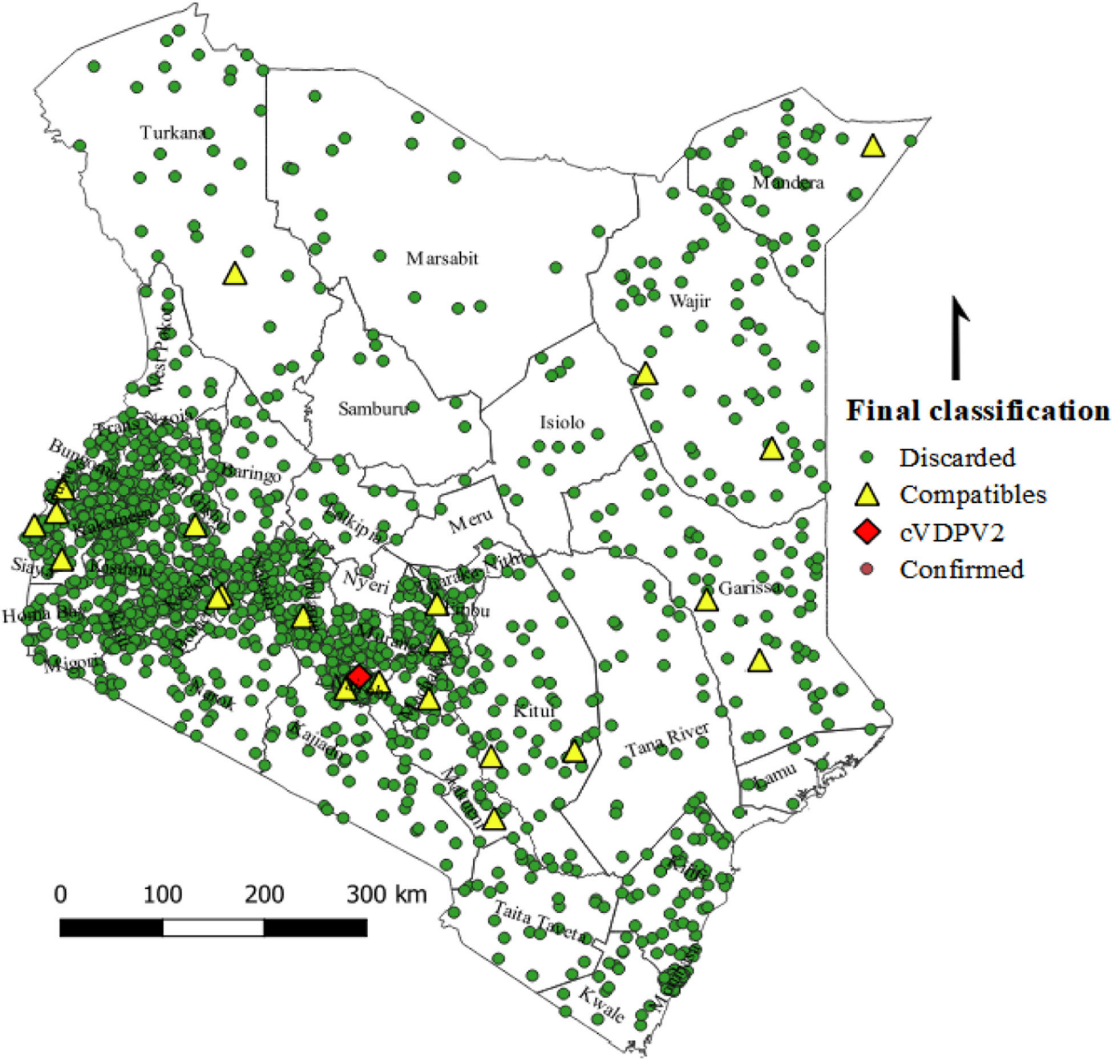
Spatial distribution of AFP cases by final virological classification, Kenya, 2016–2018

A total of 1706 cases of AFP and 160 contacts were reported from children under 15 years of age from 2016 to 2018. The mean age of children was 5.17 years (+ 4.6 years) and children under five accounted for 1085 (63.6%). The male to female ratio is s 1:1.2. However, data on sex for 22 (1.3%) AFP cases was missing (Table 1). None of the detected cases was classified as poliomyelitis, but the NPEC classified 23 (1.35%) AFP cases as polio compatible according to the WHO virological classification flowchart [28] (Fig. 2).

**Table 1:** Demographic characteristics, vaccination and clinical history of AFP cases, Kenya, 2016–2018

Demographic characteristics and clinical history	Year of onset of paralysis		
	2016 N (%)	2017 N (%)	2018 N (%)
Age			
0–5	365 (64.4%)	284 (62.0%)	436 (64.0%)
6–9	90 (15.9%)	77 (16.8%)	127 (18.6%)
10–15+	112 (19.8%)	88 (19.2%)	103 (15.1%)
Missing	0 (0.0%)	9 (2.0%)	15 (2.2%)
Sex			
Male	322 (56.8%)	221 (48.3%)	376 (55.2%)
Female	240 (42.3%)	210 (45.8%)	305 (44.8%)
Missing	5 (0.9%)	17 (5.9%)	0 (0.0%)
Vaccination status			
Zero dose	1 (0.2%)	2 (0.4%)	12 (1.8%)
1–2 doses	38 (6.7%)	58 (12.7%)	52 (7.6%)
3+ doses	511 (90.1%)	398 (86.9%)	594 (87.2%)
Missing	17 (3.0%)	0 (0.0%)	23 (3.4%)
Clinical history			
Fever at onset of paralysis	394 (69.5%)	285 (62.2%)	420 (61.7%)
Paralysis progressed within 3 days	330 (58.2%)	275 (60.0%)	371 (54.5%)
Asymmetrical paralysis	529 (93.3%)	157 (34.3%)	603 (88.5%)
Flaccid sudden onset of paralysis	340 (60.0%)	392 (85.6%)	172 (25.3%)

Table 1 shows that sudden onset of paralysis was reported in 904 (53.0%) of the AFP cases, paralysis progression within 3 days of onset in 976 (57.2%) cases, and 1099 (64.5%) AFP cases reported fever at the onset of paralysis. Majority of the cases (88.1%) had received the recommended three and above doses of OPV (Table 1). One hundred sixty AFP contacts were identified and stool specimens collected from them.

### AFP surveillance performance

**Fig. 3:**
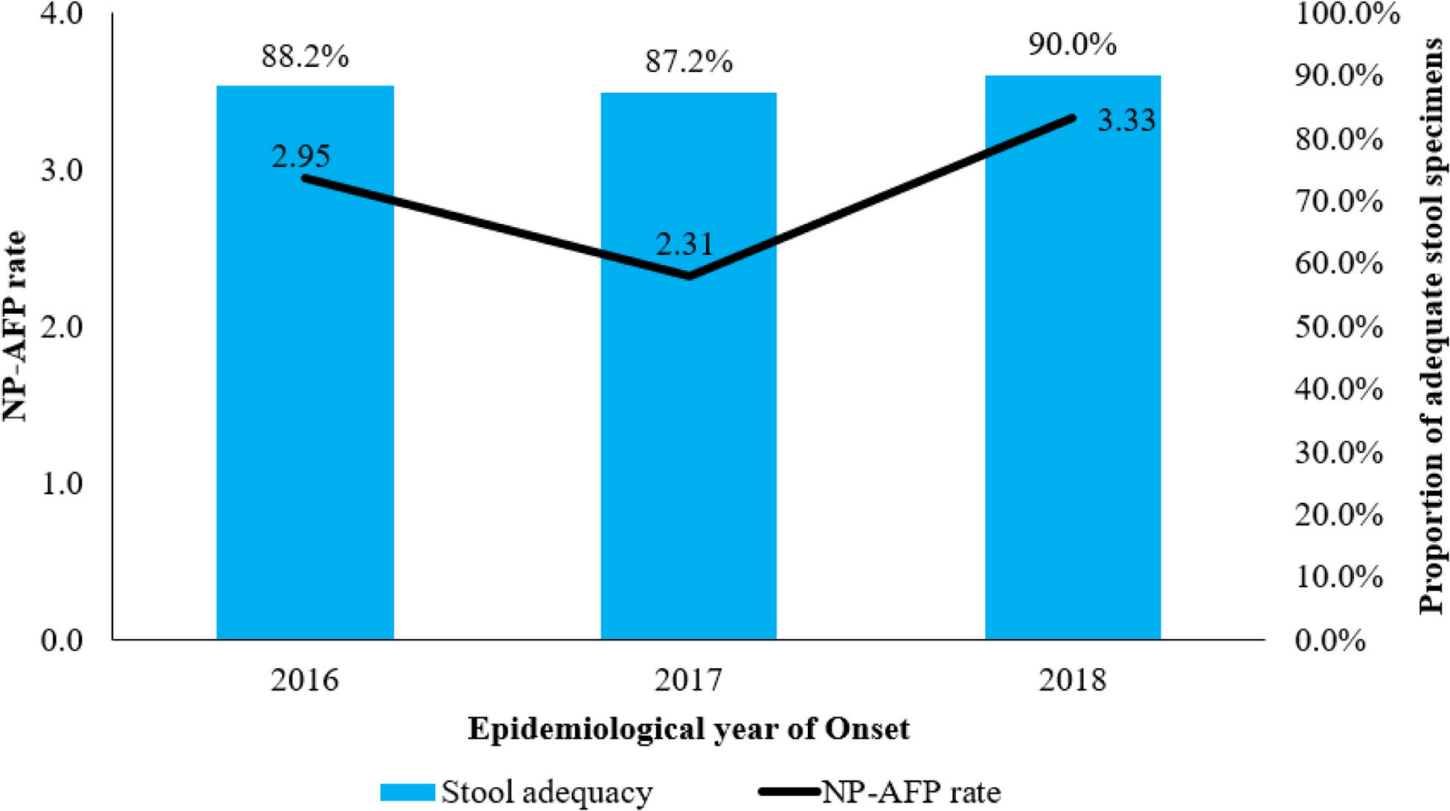
Mean NP-AFP rate and stool adequacy, Kenya, 2016–2018

**Fig. 4:**
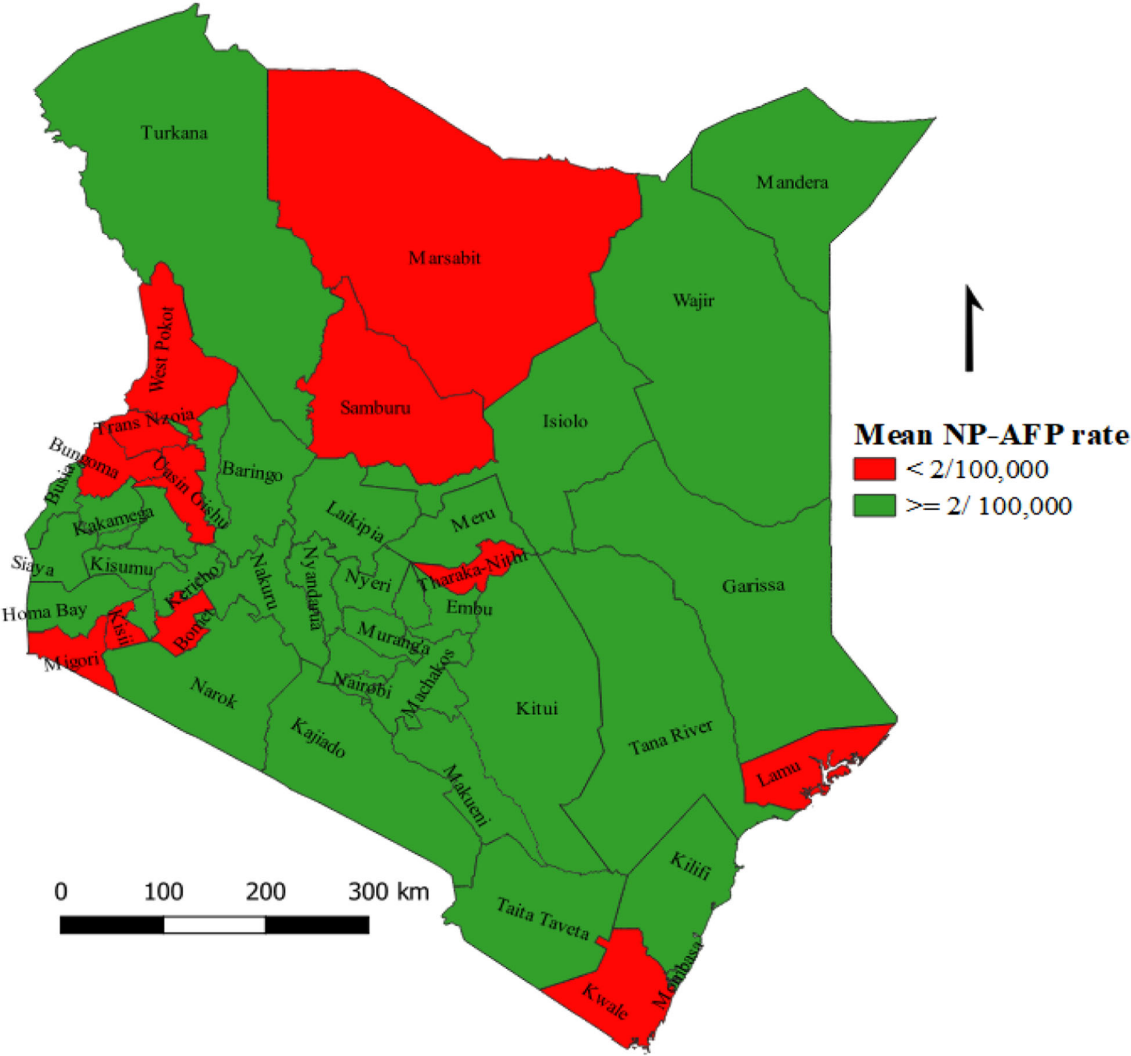
Mean NP-AFP rate by county, Kenya, 2016–2018

The mean NP-AFP rate during the study period was 2.87 per 100,000 children under 15 years; 2.95/ 100, 000 in 2016, 2.31/ 100,000 in 2017 and 3.33/ 100,000 in 2018 (Fig. 3). The sub-national mean NP-AFP rate ranged from 1.07/ 100,000 in Lamu county to 8.54/ 100,000 in Garissa county. Of the total 47 counties, 35 (74.5%) met the WHO minimum target of 2/ 100,000 children under 15 years (Fig. 4). Overall, a 20% increment was observed in the number of AFP cases detected between 2016 and 2018 at national level. Despite this progress at the national level, a declining trend was observed in Homa-Bay, Kwale, Makueni, Nyamira, Vihiga, Uasin-Gishu, Turkana, Trans-Nzoia, Samburu and Nyeri counties.

**Fig. 5:**
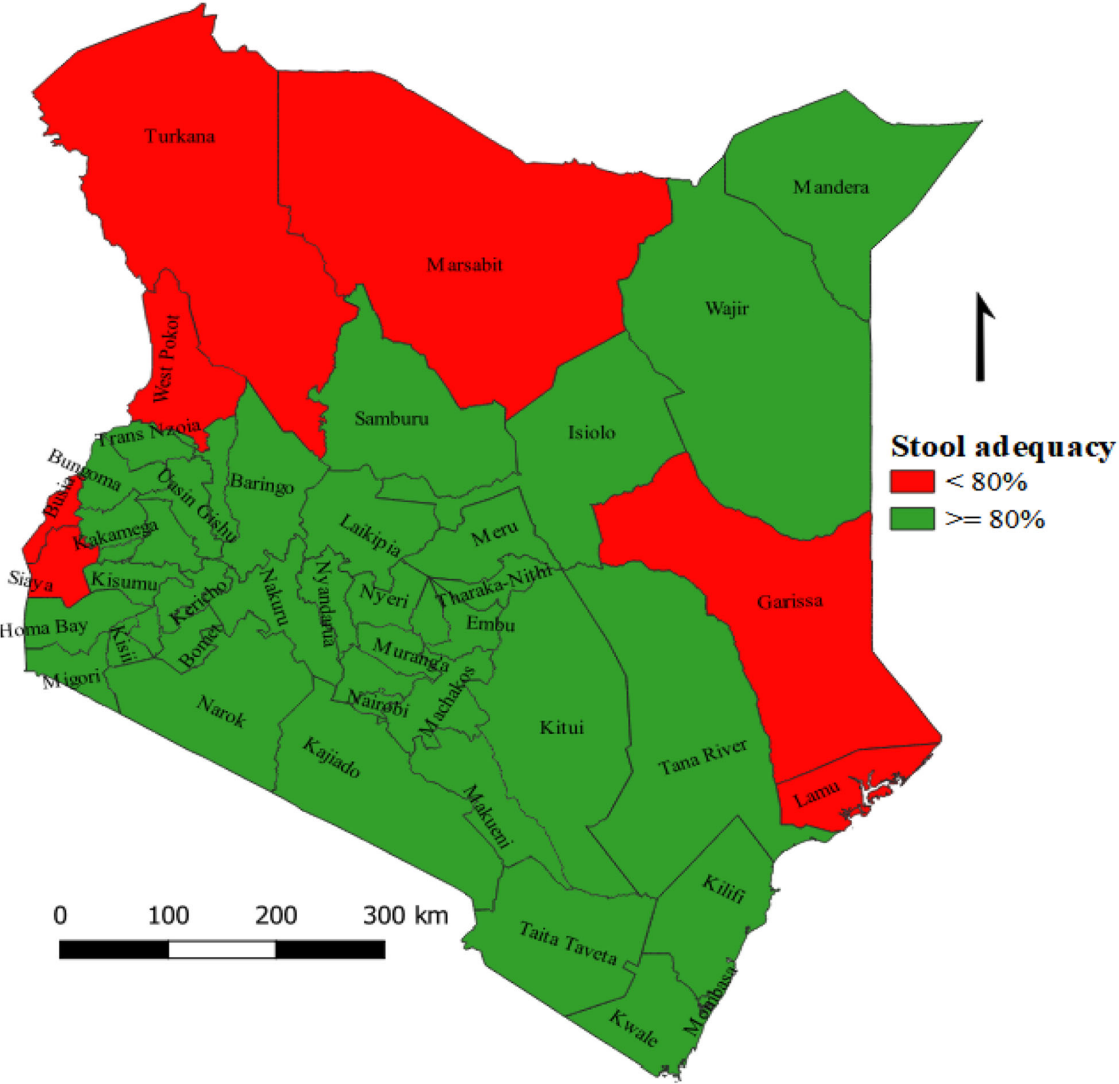
Mean stool adequacy by county, Kenya, 2016–2018

Between 2016 and 2018, adequate stool specimens were collected for 1512 (88.6%) AFP cases. The proportion of AFP cases with adequate stool specimens was constantly above the minimum target of 80% and above nationally, 88.2% in 2016, 87.2% in 2017 and 90.0% in 2018 (Fig. 3). Sub-national mean proportion of adequate specimens ranged from 66.7% in Lamu to 100.0% in Kericho, Kwale, Samburu, Tana-River and Tharaka-Nithi counties. On average, 7 (14.9%) counties; Lamu, West-Pokot, Marsabit, Turkana, Siaya, Garissa and Busia, failed to meet the minimum target of > 80% and above (Fig. 5) during the study period.

**Fig. 6:**
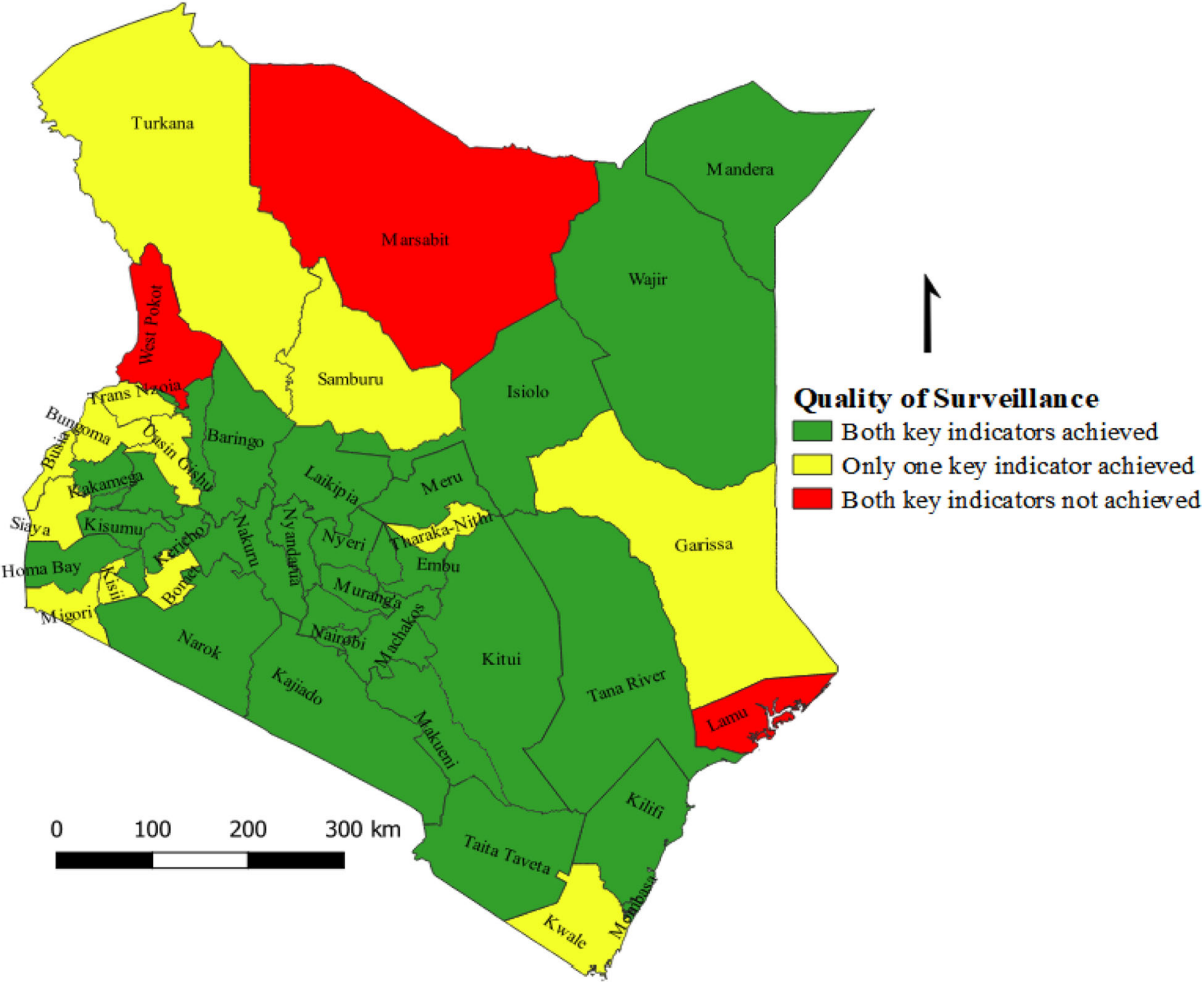
Mean **c**ombined NP-AFP rate and stool adequacy, Kenya, 2016–2018

On average, more than half (66.0%) of the counties in Kenya achieved targets for the combined AFP surveillance performance indicator (NP-AFP rate and stool adequacy) during the period reviewed. Whilst most of the remaining counties had achieved the set targets for one of the key surveillance indicators, Lamu, West-Pokot and Marsabit failed on both key surveillance indicators on average during the study period (Fig. 6).

## Discussion

The present study aimed to describe AFP surveillance performance in Kenya from 2016 to 2018 based on the WHO recommended performance indicators for AFP surveillance. It demonstrated the mean national performance disaggregated by counties and provided evidence on performance of key AFP surveillance indicators improved markedly throughout the study periods.

The main goal of any AFP surveillance system is to detect, investigate, report, disseminate and promptly implement control measures in a timely manner for indigenous and imported cases of WPV or cVDPV [29]. This goal is also expandable to certify polio free status [7, 19]. No case of poliomyelitis was reported from 2016 to 2018. However, a cVDPV type 2 was isolated from an environmental sample in Nairobi County in March 2018. The Poliovirus isolated had similar nucleotide sequence structure to poliovirus isolated in Somalia. This highlights that there is a potential for Poliovirus importation in Kenya especially from polio outbreak neighboring countries thereby justifying the need for strengthening AFP surveillance [7, 30]. As part of the outbreak response, five successive rounds of polio SIAs were conducted in 12 high-risk counties representing about 25% of all counties in Kenya. The SIAs were conducted between May to September 2018. Active surveillance for AFP cases was conducted during the SIAs.

Our finding showed that majority of the AFP cases were below the age of 5 years (59 months). This is in agreement with findings from previous studies in Ghana, Iran, Nigeria and India [8, 24, 31]. On the contrary, a study in Italy [32] highlighted a smaller percentage of AFP cases occurring among under five children.

Our findings also give credence to the differential of reported AFP cases by gender. Our results revealed that the mean proportion of AFP cases was higher in boys (55.1%) as compared to girls (44.9%), although the difference was not statistically significant. The result from this study is in line with previous studies conducted in Ghana, Iran, Ethiopia and Italy [8, 24, 32, 33].

The current study also evidenced that higher percentage of children had developed fever at onset of paralysis, which progressed within 3 days and was asymmetric. Authors believe that this could be accredited to appropriate implementation of AFP case definition during case investigation to determine if the reported case meets the case definition for AFP or not. Different studies across the globe have upheld the same finding [24, 33, 34].

Two types of polio vaccine are used in polio eradication efforts [8, 35]; the Inactivated Polio Vaccine (IPV) which is administered through an injection and the Oral Polio Vaccine (OPV) which is administered orally; it is live but attenuated (weakened) virus developed by Sabin [36]. The Oral Polio Vaccine is the main vaccine towards polio eradication. When OPV is administered, the weakened vaccine-virus replicates in the intestine and enters into the bloodstream, triggering a protective immune response. During replication process, some of the vaccine-virus may genetically mutate from the original attenuated strain and become neurovirulent. The neurovirulent virus from the polio vaccine is referred to as Vaccine-Derived Poliovirus (VDPV), which is able to cause paralysis [20]. The VDPV is able to circulate in the community, known as circulating Vaccine-Derived Poliovirus (cVDPV) [20], most likely due to weak AFP surveillance, suboptimal routine immunization coverage, and constant movement of populations [20] in cross-border high-risk areas. In the case of the IPV vaccine, the virus is inactivated. Therefore, it cannot regain virulence after it has been administered to a child. The Inactivated Polio Vaccine is not a replacement for OPV but rather used in addition to OPV to protect against polio serotype 2 which is not in the current OPV and also strengthen immunity against the other two serotypes. A vast majority of the AFP cases had received the recommended three and above doses of OPV. It is possible that the presented proportion of AFP cases that is said to have received at least three doses of OPV might have been under- or overestimated by recall bias from parents or caregivers of the children whose immunization cards could not be traced [24, 37]. In addition to the routine immunization program, the high polio vaccination coverage in reported AFP cases is attributable to high quality polio SIAs conducted sixth times with greater emphasis on high risk and underserved population sub-groups. High immunization coverage through routine immunization and SIAs boost herd immunity and, disrupts chain of infections and silent circulation of poliovirus [38–40]. The findings of our study highlight that Kenya has sensitive and quality AFP surveillance system capable of detecting and reporting Poliovirus cases. The health workers’ industrial action in 2017 might have greatly affected the performance of AFP surveillance and routine immunization activities as depicted by lower performance in 2017 in comparison with 2016 but better performance in 2018 that in 2016. It is worth noting that the minimum NP-AFP rate and stool adequacy targets set by the WHO were surpassed at the national level in all the 3 years analyzed. Furthermore, drill-down analysis of the mean NP-AFP rate by counties showed heterogeneous performances. Some counties such as Tana-River, Wajir, Isiolo and Garissa outperformed while others for instance Lamu, West-Pokot, Migori and Trans-Nzoia were least performers.

The findings of the study showed that the mean proportion of AFP cases with adequate specimens surpassed the WHO recommended target of 80% and above from 2016 through 2018. Like, NP-AFP rate, there were disparities in stool adequacy performance among counties. Lamu, West-Pokot, Marsabit, Turkana, Siaya, Garissa and Busia counties were unable to meet WHO recommended target for collection of two adequate specimens within 14 days of onset of paralysis. As important as case detection, laboratory analysis is crucial to the confirmation of the poliovirus [28]. Thus, the stool specimens collected from AFP cases need to be of a quality that meets minimum laboratory requirements. Subsequently, with high stool adequacy rates coupled with no poliovirus isolation during the study period, it is reasonable to believe that there was no wild poliovirus circulating in the country during the study period, although the risk of poliovirus importation from neighboring countries with relatively weaker health systems remains high due to continued influx of refugees and general movement of people.

In our study, we noted that the NPEC classified 23 AFP cases as polio compatible. The 23 polio compatible cases lent an unprecedented opportunity to cluster investigation of this subgroup of AFP cases by geographical area and time [1], which was very important towards polio eradication activities [31]. Cluster investigation and additional passive and active case searches conducted verified that there were no missed polio cases and dismissed the presence of circulation with confidence.

## Conclusions

Kenya’s AFP surveillance system surpassed the WHO recommended minimum targets for both non-polio AFP rate and stool adequacy during the period studied. A substantial increment was observed in detection rate. Most counties achieved targets for both key surveillance indicators. Health worker awareness on AFP surveillance and active case search should be strengthened in all aspects of polio eradication activities with greater emphasis on high-risk counties that consistently failed to meet targets on both sensitivity and quality indicators. Documentation of clinical diagnosis for AFP cases and 60 days follow up needs to be improved. AFP surveillance indicators should be analyzed at the sub-county level to uncover underperformance that might be masked by county level analyses including “Silent” districts.

## Data Availability

The data used for this study can be accessed with justifiable request.

## Abbreviations

AFP: Acute Flaccid Paralysis
cVDPV2: circulating Vaccine-derived Poliovirus type 2
GPEI: Global Polio Eradication Initiative
IPV: Inactivated Polio Vaccine
NP-AFP: Non Polio Acute Flaccid Paralysis
NPEC: National Polio Expert Committee
OPV: Oral Polio Vaccine
SIAs: Supplemental Immunization Activities
WHO: World health Organization
WPV: Wild Poliovirus
